# Incidence of adult Huntington's disease in the UK: a UK-based primary care study and a systematic review

**DOI:** 10.1136/bmjopen-2015-009070

**Published:** 2016-02-22

**Authors:** Nancy S Wexler, Laura Collett, Alice R Wexler, Michael D Rawlins, Sarah J Tabrizi, Ian Douglas, Liam Smeeth, Stephen J Evans

**Affiliations:** 1 Department of Neurology and Psychiatry, Columbia University, New York, New York, USA; 2 Hereditary Disease Foundation, New York, New York, USA; 3 Department of Medical Statistics, London School of Hygiene and Tropical Medicine, London, UK; 4 Department of Non-Communicable Disease Epidemiology, London School of Hygiene and Tropical Medicine, London, UK; 5 Department of Neurodegenerative Diseases, Institute of Neurology, University College London, London, UK

**Keywords:** EPIDEMIOLOGY, GENETICS

## Abstract

**Objectives:**

The prevalence of Huntington's disease (HD) recorded in the UK primary care records has increased twofold between 1990 and 2010. This investigation was undertaken to assess whether this might be due to an increased incidence. We have also undertaken a systematic review of published estimates of the incidence of HD.

**Setting:**

Incident patients with a new diagnosis of HD were identified from the primary care records of the Clinical Practice Research Datalink (CPRD). The systematic review included all published estimates of the incidence of HD in defined populations.

**Participants:**

A total of 393 incident cases of HD were identified from the CPRD database between 1990 and 2010 from a total population of 9 282 126 persons.

**Primary and secondary outcome measures:**

The incidence of HD per million person-years was estimated. From the systematic review, the extent of heterogeneity of published estimates of the incidence of HD was examined using the I^2^ statistic.

**Results:**

The data showed that the incidence of HD has remained constant between 1990 and 2010 with an overall rate of 7.2 (95% CI 6.5 to 7.9) per million person-years. The systematic review identified 14 independent estimates of incidence with substantial heterogeneity and consistently lower rates reported in studies from East Asia compared with those from Australia, North America and some—though not all—those from Europe. Differences in incidence estimates did not appear to be explained solely by differences in case ascertainment or diagnostic methods.

**Conclusions:**

The rise in the prevalence of diagnosed HD in the UK, between 1990 and 2010, cannot be attributed to an increase in incidence. Globally, estimates of the incidence of HD show evidence of substantial heterogeneity with consistently lower rates in East Asia and parts of Europe. Modifiers may play an important role in determining the vulnerability of different populations to expansions of the HD allele.

**Strengths and limitations of this study**

- The study provides the most reliable estimates of the incidence of Huntington's disease (HD), in the UK, between 1990 and 2010.
- The study also provides a comprehensive and contemporary review of the published incidence of HD globally.
- The study of the incidence of HD in the UK relies on new diagnoses of HD being reported in primary care records.
- The systematic review does not attempt a quantitative assessment of the quality of the included studies.

Huntington's disease (HD) is an autosomal-dominant neurological disorder.^1^ ^2^ Located on chromosome 4p16.3, the normal form of the gene contains up to 38 trinucleotide repeats. The abnormal form of the gene has from 40 to 125 trinucleotide repeats.^3^ The HD gene codes for a protein called huntintin. The abnormal form of the gene codes for a toxic protein. The gene can expand during transmission, possibly leading to different rates of incidence and prevalence in different populations.

HD usually presents in early to middle life with abnormal movements including chorea, dystonia and rigidity.^1^ ^2^ Patients may also suffer severe psychiatric complications including hallucinations, delusions, obsessive-compulsive disorder, depression and bipolar disorder.^2^ They contend with progressive cognitive loss.^1^ ^2^ It is uniformly fatal over a 10–20-year decline. Aspiration pneumonia and suicide are common causes of death.^2^

A previous study by us has shown^4^ that the prevalence of HD, as diagnosed and recorded in primary care records, has increased from 5.4 (95% CI 3.8 to 7.5) per 100 000 in 1990 to 12.3 (95% CI 11.2 to 13.5) per 100 000 in 2010. In order to explore the basis for this unexpected finding, we have undertaken a study of the incidence of HD in the UK over the same time period.

To place our own findings in context we have also undertaken a systematic review of published estimates of the incidence of HD. A previous systematic review of the incidence (as well as prevalence) of HD was published in 2012^5^ and identified eight studies. That review only included studies carried out between 1985 and 2010 on the grounds that before 1985 MRI was not routinely in clinical use. Since the diagnosis of symptomatic (manifest) HD is essentially a clinical one, and not dependent on imaging, the present review attempted to identify all published estimates of the incidence of HD published between 1950 and 2014. We sought to examine the heterogeneity between these estimates and, in particular, the extent to which any observed differences between populations might be explained by methods used for case finding and diagnosis.

## Methods

### UK population-based estimates of the incidence of HD

#### Study design and setting

The Clinical Practice Research Datalink (CPRD), formerly the General Practice Research Database (GPRD), is a computerised database of anonymised longitudinal medical records from primary care that has been collected for over 25 years. The CPRD is assembled from the electronic health records of patients registered with around 625 contributing general practices and with over 5 million patients currently enrolled (representing approximately 8% of the UK population). The practices are broadly representative of those in the UK in geographical distribution, practice size, age and sex as well as ethnicity and body mass index of registered patients.^6^ Each individual patient is assigned a unique identification number. No information from their medical records, allowing identification of individual patients, is included in the database. The data are therefore entirely anonymous to investigators. It is also important to appreciate, for the benefit of those unfamiliar with the UK's National Health Service, that patients requiring specialist services must be referred by their general practitioner (GP). The referring GP will invariably be informed of the results of all investigations and the diagnosis.

CPRD includes the complete diagnostic and prescribing information for each registered patient. When patients newly register with a contributing practice, major past and existing diagnoses are recorded in their medical records and are included in the research database. However, the dates of onset and of past diagnoses are not always accurately recorded. In particular, some diagnoses that occurred in the past may be recorded without a date or as occurring at, or shortly after, the date of registration.

Morbidity in UK primary care is recorded using Read codes Clinical Terms V.3.^7^ At both practice and individual patient levels, the data are subject to a range of quality checks prior to being made available for research purposes.^8^ The quality of the data has been found to be high in a large number of independent validation studies.^9^

The potential funders of the study played no part in its design, analysis or interpretation.

#### Participants and variables

The source population was all patients aged 21 years or more who were registered with general practices contributing to the CPRD between 1990 and 2010. The age of 21 was used to distinguish adult form of HD from the very rare juvenile form of the condition.^1^ ^2^ Eligible cases were defined as persons with one or more recorded diagnoses of HD or Huntington's chorea in their medical records. The Read codes used to identify cases of HD were F134.00 (Huntington's chorea) and Eu02200 (dementia in HD).

For each general practice record, the observation period for the study began as the later of two dates: either the study start date (1 January 1990) or the date at which the practice started contributing research standard data to the CPRD. The end of the observation period was the earlier of two dates: the last date for which the practice contributed data to the CPRD, or the study end date (31 December 2010). Individual patients were included in denominators only during times within the observation period that they were registered with a practice contributing data to the GPRD and were aged at least 21 years.

Incident patients were defined as all those with a first record of an HD diagnostic code, during the observation period, but with two additional criteria. (1) The patients’ first recorded HD diagnosis was required to have occurred at least 12 months after their first entry in the database. In other words, at least 12 months were required to have elapsed since their registration date. (2) Patients’ were required to have at least two recorded contacts with their contributing practice prior to their HD diagnosis. These additional criteria helped avoid including prevalent cases as if they were incident ones.

#### Statistical methods

Incidence was calculated from the ratio of number of persons with a new recorded diagnosis of HD for each year from 1990 to 2010, divided by the total number of persons in the database for that year, who had also had at least 1 year in the database and were aged at least 21 years. Binomial CIs were calculated. In estimating the incidence in age bands, annual incidence estimates were averaged and approximate (binomial) 95% CIs calculated. All incidence rates are expressed per million person-years.

### Systematic review of studies of the incidence of HD

The criteria for inclusion in the systematic review were that a study should be based on a defined population and that it should provide information about the number of new HD diagnoses made within one or more specific time frames. No study was excluded by virtue of its date, the ages studied, or because of the approach taken to either case ascertainment or the diagnosis of HD. The search dates covered the period January 1950 to December 2013. There were no language restrictions.

Relevant publications were sought as follows:

1. Search of MEDLINE and EMBASE (see web extra annex 1). Studies fulfilling the inclusion criteria, but published only as abstracts, were considered for inclusion.
2. Scrutiny of the references quoted in reviews of the epidemiology of HD.^10–17^
3. Examination of the reference lists in publications meeting the inclusion criteria.

After the removal of duplicates, the full texts of the remaining articles were examined. The relevant details of those meeting the inclusion criteria were transcribed independently (by ARW and MDR) and entered into data extraction forms. Disagreements due to minor transcription errors were resolved by consensus. The data extraction forms recorded:

- The full reference;
- The geographical location and year(s) of the study;
- The relevant population size;
- The number of patient-years used in the estimation(s) of incidence;
- The method(s) of case ascertainment;
- The method(s) of diagnosis;
- The number of new patients with HD during the year(s) of study;
- The mean age at diagnosis, where provided, of patients with HD together with the age range or the 95% CIs;
- The estimate(s) of incidence with (if calculated) their 95% CIs.

10.1136/bmjopen-2015-009070.supp1 Supplementary data

In some instances, where numerical data were lacking, the number of patient-years was estimated by back extrapolation. Where no 95% CIs were provided, these were calculated from the available data. Reporting follows, where appropriate, the PRISMA guidelines.^18^

#### Statistical methods

Incidence rates and their 95% CIs were recalculated from the original reports from the number of new HD diagnoses divided by the number of person-years. In some instances, where either the number of cases or the number of person-years was not quoted, these were estimated by back extrapolation. The degree of heterogeneity was estimated from the I^2^ test.^19^

## Results

### The incidence of adult HD in the UK

A total of 393 incident cases of HD were identified from the CPRD database between 1990 and 2010 from a total population of 9 282 126 persons (corresponding to 54 907 468 person-years). The incidence rates, in 5–6-year bands (table 1), showed no significant changes between 1990 and 2010. The apparent fluctuations between 1990 and 1994 (see web extra annex 2) are likely to be due to sampling error as both the number of incident cases, and the denominators, were relatively small during these years. The average incidence rate of HD, during the entire period, was 7.2 (95% CI 6.5 to 7.9) per million patient-years. Rates for females (7.1, 95% CI 6.1 to 8.10) and males (7.3, 95% CI 6.3 to 8.4) were similar.

**Table 1:** Average incidence rates and age of onset of HD 1990–2010

Years	Incident cases	Denominators (patient years)	Incidence per million patient years (95% CIs)	Mean age of onset in years (SD and IQR)
1990–1996	56	6 778 613	8.26 (6.24 to 10.73)	51.5 (13.9 40 to 61)
1997–2003	138	18 533 173	7.45 (6.26 to 8.80)	53.1 (13.7 44 to 64)
2004–2010	199	29 522 583	6.7 (5.84 to 7.75)	52.1 (16.4 40 to 65)

HD, Huntington's disease.

**Figure 1:**
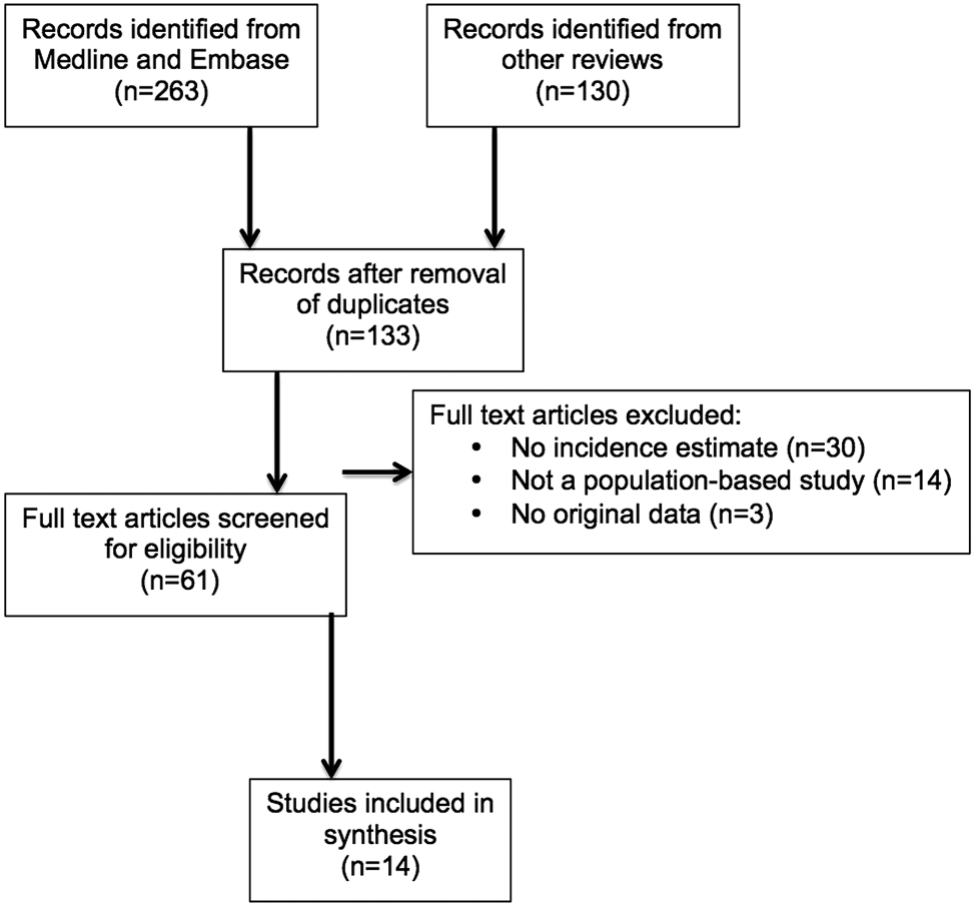
Flow diagram of search strategy.

The mean age at diagnosis was 52.4 years (SD 15.1; IQR 42–64 years; see figure 1). It was almost identical in females (mean 52.4; SD 15.2; IQR 42–64 years) and males (52.4; SD 15.1; IQR 41–64 years). The ages of onset are shown in table 1 and do not show any significant change over the period of the study. The average annual incidence rates in relation to the age of onset are shown in table 2.

**Table 2:** Incidence and age of onset

	Incidence per million patient years (95% CIs)		
Age of onset (years)	1990–1996	1997–2003	2004–2010
<40	7.9 (4.4 to 13.1)	5.6 (3.7 to 8.1)	6.6 (4.9 to 8.7)
40–49	13.2 (7.0 to 22.6)	11.8 (8.0 to 16.8)	10.8 (8.0 to 14.3)
50–59	14.8 (7.6, 25.8)	13.1 (9.0 to 18.4)	7.2 (4.8 to 10.4)
>60	10.0 (5.7 to 16.2)	10.7 (7.8 to 14.3)	10.3 (8.1 to 13.0)

### Systematic review of the incidence of HD

The numbers of studies initially identified, screened, subjected to full-text review and included in the final synthesis are shown schematically in figure 1. Additional details of the included and excluded in studies are available in the web extra tables 2–5. We identified 14 published studies (figure 1) which, together with the results of the present study, provided 15 investigations into the incidence of HD for inclusion in our systematic review (table 3).

**Table 3:** Incidence studies from the systematic review

Study ID	Location	Study year(s)	Incident cases	Patient-years	Incidence per 1 000 000 person-years (95% CIs)	Age of onset	Comments
Eastern Asia							
Chen 1968^20^	Guam	1960–1966	0	265 825	0.00 (0 to 13.9)	Not stated	
Chang 1994^23^	Hong Kong, China	1984–1991	20	43 520 000	0.46 (0.28 to 0.71)*	37.6 (range 20–52)	
Chen 2010^24^	Taiwan	2000–2007	165	15 900 000	1.04 (0.89 to 1.21)	Not stated	Average incidence between 2000 and 2007
Australasia							
McCusker 2000^31^	NSW, Australia	1991 and 1996	1991=26 1996=39	1991=5 732 031 1996=6 038 969	1991=4.5 (3.0 to 6.7) 1996=6.5 (4.6 to 8.8)	47.9 (SD 13.7)	
Europe							
Palo 1987^25^	Finland	1980s	2†	4 900 000	0.2 to 0.4 (0.02 to 1.3)*	Not stated	
Govoni 1988^27^	Ferrara, Italy	1971–1987	14	6 032 096‡	1.1 (0.4 to 2.3)	Not stated	
Ramos-Arroyo 2005^30^	Navara and Basque, Spain	1994–2002	111	21 165 000	4.7 (4.5 to 6.3)*	43.7 (SD 15)	
Mercy 2008^21^	Cambridge UK	2000–2006	9	453 600	8.0 (2.0 to 23)	Not stated	Restricted age range (45–64 years)
Panas 2011^29^	Greece	1995–2008	48†	10 964 020	4.38 (3.23 to 5.50)	44.0 (SD 12.9)	
Sackley 2011^28^	UK	2004–2008	85	14 713 708	5.71 (4.45 to 7.07)	48.3 (SD14.)	Average incidence between 2004 and 2008
Sveinsson 2012^26^	Iceland	1988–2007	8	5 714 285‡	1.4 (0.6 to 2.8)*	51 (range 28–68)	
Douglas 2013^22^	UK	1990–2010	12	17 142 857	0.70 (0.36 to 1.22)	Median=15 (range 5–20)	Restricted to juvenile HD
Current study	UK	1990–2010	393	54 907 468	7.2 (6.5 to 7.9)	52 years (SD 16)	Also includes annual incidence rates1990–2010
North America							
Kokman 1994^32^	Minnesota, USA	1950–1989	10	4 134 000‡	Definite=3.0 (1.0 to 5.0) Definite+probability=5.0 (3.0 to 9.0)	Not stated	.
Almqvist 2001^33^	British Columbia, Canada	1996–1999	110	16 058 394‡	6.9 (5.7 to 8.3)*	46.9 (SD 13.7)	Author states population is ‘approximate’

*95% CIs not included in the published report but estimated for this review.

†Numbers of patients with HD calculated by back extrapolation.

‡Patient-years calculated by back extrapolation.

HD, Huntington's disease; NSW, New South Wales.

Estimates of the incidence of HD (table 3) ranged from 0 (95% CI 0 to 97.0)^20^ to 8.0 (95% CI 2.0 to 23) per million person-years,^21^ although the latter study was conducted among a restricted age range (45–64 years). It is clear from this table that there is marked heterogeneity (I^2^=98.5%, 95% CI 98.2% to 98.6%) across studies, even after the omission of the two age-specific estimates.^21^ ^22^ Lower estimates of incidence were consistently reported for studies conducted in the East Asia region including Guam,^20^ Hong Kong^23^ and Taiwan^24^ compared with most of those undertaken in Europe, North America and Australia. There is, though, heterogeneity between European estimates of incidence. Studies conducted in Finland,^25^ Iceland^26^ and the Ferrara region of Italy^27^ show incidence estimates of around one per million person-years or less. Other studies carried out in UK,^21^ ^28^ as well as the results reported here, suggest incidence rates of around 7–8 per million patient years (table 4). Estimates undertaken in Greece,^29^ the Basque region of Spain,^30^ Australia^31^ and North America^32^ ^33^ are comparable to those in the UK including the present study. The low incidence of the juvenile form of HD,^22^ in the UK, is to be expected in the light of the known low prevalence of this very rare form of HD.

**Table 4:** Sources of cases and diagnostic criteria used in the included studies

Study ID	Location	Source of cases	Diagnostic criteria
Asia			
Chen 1968^20^	Guam	Records of patients attending the Guam Memorial Hospital	Not stated
Chang 1994^23^	Hong Kong, China	Computer search of all major hospitals. Announcement in Hong Kong Medical Association Newsletter asking for information about known or suspected cases. Enquiry of all neurologists and psychiatrists in Hong Kong	All patients examined by a neurologist plus a psychiatrist. Diagnosis based on positive family history plus insidious progressive disorder with chorea, cognitive impairment and often psychiatric disturbance. Positive CT scan with caudate atrophy considered to be ‘supportive’ of an HD diagnosis
Chen 2010^24^	Taiwan	Outpatient and inpatient claims from the National Health Insurance Research Database	Search of National Health Insurance Research Database for ICD-9 code 333.4
Australasia			
McCusker 2000^31^	NSW, Australia	Records of the NSW HD Service. Records of the major general and chronic psychiatric hospitals in NSW. Questionnaires to adult and paediatric neurologists, psychiatrists, genetic counsellors and clinical geneticists	Definite: chorea or ataxia with a positive family history or expanded CAG repeat
Europe			
Palo 1987^25^	Finland	Systematic search of all university, central, general and central psychiatric hospitals	Not stated
Govoni 1988^27^	Ferrara, Italy	Records of the neurology clinics of Ferrera and Bologna, civil records, records of the psychiatric institutions, records of public and private geriatric nursing homes	Combination of a positive family history, choreiform movements, mental deterioration
Ramos-Arroyo 2005^30^	Navara and Basque, Spain	Referrals to the Medical Genetics Laboratory of the Hospital Virgen del Camino, Pamplona, Spain, for diagnostic testing for HD between 1993 and 2002. Also searched for additional patients from the Basque country who might have been referred to other HD diagnostic genetic centres in Spain. In addition, patients who underwent presymptomatic testing and became symptomatic within the study period were also included	Definite=typical clinical features plus <36 CAG repeats plus positive family history Suspect=without positive family history
Mercy 2008^21^	Cambridge UK	Attendees/referrals to Addenbrooks’ Hospital memory/early dementia clinic	UHDRS >5
Panas 2011^29^	Greece	Records of the Laboratory of Neurogenetics, Athens (the only neurogenetics lab in Greece)	Neurological examination including the UHDRS plus CAG repeat length in a subset of patients
Sackley 2011^28^	UK	Using THIN primary care research database the authors identified Read codes for HD	Based on recorded diagnosis
Sveinsson 2012^26^	Iceland	Medical records and hospital discharge diagnoses of all hospitals including records of neurological, psychiatric and genetic departments. Information from practising neurologists and selected GPs. Information from family members	Hyperkinetic movement disorder plus psychiatric symptoms plus progressive cognitive decline plus a positive family history or positive DNA analysis
Douglas 2013^22^	UK	Primary care National Health Service electronic health records	As recorded in patients’ electronic health records (Read codes F134.00 and Eu2200)
Current study	UK	Primary care National Health Service electronic health records	As recorded in patients’ electronic health records (Read codes F134.00 and Eu2200)
North America			
Kokman 1994^32^	Minnesota, USA	Scrutiny of records of hospitals, nursing homes, private practitioners, state psychiatric hospital	Definite HD=documented record of progressive choreiform movement disorder; evidence of autosomal dominant inheritance; progressive cognitive, behavioural, and/or emotional dysfunction. Probable HD=2 out of 3 of the above criteria
Almqvist 2001^33^	British Columbia, Canada	Patients referred to Medical Genetics Laboratory/HD clinic	Patients with signs and symptoms compatible with HD and with CAG repeat lengths >36

HD, Huntington's disease; GP, general practitioner; ICD, International Classification of Diseases; NSW, New South Wales; THIN, The Health Improvement Network; UHDRS, United Huntington's Disease Rating Scale.

The approaches used in case ascertainment, and the criteria for accepting a diagnosis of HD among the included studies, are shown in table 4. A variety of methods were used in identifying patients with HD and, to a lesser extent, in the diagnostic criteria each study adopted. However, this variability is inadequate to explain the marked differences in the heterogeneity of incidence worldwide.

## Discussion

### UK estimate of incidence

The absence of any consistent change in the incidence of HD in the UK, between 1990 and 2010, is in marked contrast to the substantial increase in the prevalence of HD over the same period. Evans *et al*^4^ offered a number of possible explanations for the apparent rise in prevalence. These included (1) an increase in incidence; (2) an increase in the diagnosis of HD as a result of the availability of a genetic test permitting physicians to diagnose HD in patients with atypical symptoms; (3) an increase in the willingness of GPs to record a diagnosis of HD in patients’ records; (4) or an increase in the longevity of those with manifest HD as a consequence of the general population trend as well as the result of better symptomatic treatment.

Using the same database and during the same time period, our present study shows that the increase in prevalence of HD previously reported^4^ does not appear to be explained by a rise in incidence. More reliable diagnoses of HD are also unlikely to be an explanation because, if this had been the case, we should have observed an apparent increase in incidence. It is possible that patients with a prior diagnosis of HD are more likely to register with a practice contributing to the CPRD. We doubt that this is a viable explanation and, anyway, it should also be reflected by an increased incidence.

There are two remaining possibilities for this surprising rise in prevalence in the face of a constant incidence: (1) GPs are now more willing to include an HD diagnosis in the records of previously diagnosed patients potentially due to a decline in the stigma associated with the condition^34^; (2) survival has markedly improved. We are currently examining this second possibility.

### Systematic review of incidence

The present systematic review confirms the apparent heterogeneity of the incidence of HD between different populations.^5^ Studies undertaken in Eastern Asia show consistently lower estimates of incidence compared with those reported from Australasia, North America and parts of Europe. It is notable, however, that substantial heterogeneity in the incidence of HD has been reported in the European region with, as already discussed, estimates from Finland, Iceland and Northern Italy being substantially less than those reported in Spain and the UK (table 3).

Although it is possible that this observed heterogeneity in the estimates of the incidence of HD could be due to differences in the methodology of case ascertainment and diagnosis, examination of the data in table 4 suggests that this is unlikely to be the sole explanation. Indeed, the heterogeneity of the estimates of the incidence of HD is broadly consistent with the heterogeneity of estimates of the prevalence of the condition. Lower prevalence rates of HD have been noted in studies carried out in Asia compared with those among populations predominantly of European decent. The estimates of incidence among Finish and Icelandic peoples as well as Italians from the Ferrara region of Italy are also compatible with the low prevalence rates in these populations. The very low incidence of juvenile HD, in the UK^22^ (0.70, 95% CI 0.36 to 1.22 per million person-years), is also consistent with its low prevalence (6.77, 95%CI 5.6 to 8.12 per million) compared with the most recent (2010) estimate of the prevalence of adult HD (123, 95% CI 112 to 135 per million).^4^

A definitive molecular genetic test for the HD mutation has been available since the early 1990s in most developed countries. Differences in the rates of genetic testing might provide some explanation for the differences in the heterogeneity of estimates of incidence in the populations studied. This explanation also seems unlikely. For example, the overall incidence of HD in Taiwan, during the past decade, has remained consistently lower than estimates from the UK over similar time periods.

The present study suggests that, over the past two decades, the incidence of HD in the UK has remained constant despite a doubling of the prevalence of HD during this same time period. We also demonstrate that there is significant heterogeneity in the estimates of the incidence of HD carried out in other populations worldwide. Particularly low estimates of incidence in Eastern Asia, as well as parts of Europe, suggests that modifiers of the expression of the disease may play an important role in determining the propensity of populations to be vulnerable to expansions of the HD allele.^3^ ^35^ ^36^ Better understanding of the potential modifiers of expression, in different populations, may help to develop new therapeutic strategies.
