# Supplementary material for: Incidence of adult Huntington's disease in the UK: a UK-based primary care study and a systematic review
Source: BMJ Open. 2016 Feb 22;6(2):e009070. doi: 10.1136/bmjopen-2015-009070 (PMC4769413; doi:10.1136/bmjopen-2015-009070)
Supplement: Supplementary data [file bmjopen-2015-009070supp.pdf]

Annex 1 (Web extra)
Database searches

Embase

1 Huntington chorea/ (13436)
2 Huntington* chorea.tw. (1216)
3 Huntington* diseas*.tw. (9586)
4 1 or 2 or 3 (15004)
5 incidence/ (169487)
6 incidence*.tw. (496357)
7 5 or 6 (560558)
8 4 and 7 (160)

Medline

1 Huntington Disease/ (8272)
2 (Huntingt* adj2 (diseas* or chorea*)).tw. (9425)
3 or/1-2 (10898)
4 Incidence/ (148618)
5 Incidence*.tw. (427236)
6 or/4-5 (497129)
7 3 and 6 (103)

Annex 2 (web extra)
Annual incidence rates of HD 1990-2010

Year	Incident cases	Population at risk	Incidence (per million patient years)	Incidence (95% confidence intervals)
1990	1	177,201	5.64	0.14 to 31.44
1991	9	590,478	15.24	7.00 to 28.93
1992	2	887,429	2.25	0.27 to 8.14
1993	4	1,054,175	3.79	1.03 to 9.72
1994	16	1,228,009	13.03	7.45 to 8.14
1995	10	1,357,716	7.37	3.5 to 13.54
1996	14	1,483,605	9.44	5.16 to 21.16
1997	13	1,658,058	7.84	4.17 to 13.41
1998	11	1,956,579	5.62	2.81 to 10.06
1999	16	2,209,354	7.24	4.14 to 11.76
2000	21	2,574,200	8.16	5.05 to 12.47
2001	26	3,063,386	8.49	5.54 to 12.44
2002	22	3,392,342	6.49	4.06 to 9.82
2003	29	3,679,254	7.88	5.28 to 11.32
2004	33	3,905,273	8.45	5.82 to 11.87
2005	34	4,156,147	8.18	5.67 to 11.43
2006	28	4,263,980	6.57	4.36 to 9.49
2007	31	4,295,599	7.22	4.91 to 10.24
2008	19	4,330,199	4.39	2.64 to 6.85
2009	23	4,330,452	5.31	3.37 to 7.97
2010	31	4,240,933	7.31	4.97 to 10.38

Annex 3 (Web extra)
Excluded studies

Study ID	Reason(s) for exclusion
Adams 1988[w1]	Study of the age of onset of symptoms in a group of people with HD but no estimates of prevalence or incidence
Aiyesimoju 1984[w2]	No population based numerator or denominator for the incidence or prevalence of HD
Alonso 2009[w3]	No adequate data for incidence or prevalence to be estimated.
Avila-Giron 1973[w4]	No denominator. No data on prevalence
Bayulkem 1961[w5]	Denominator is numbers of hospital admissions not population based.
Bell 1934[w6]	No estimate of incidence or prevalence of HD
Brothers 1949[w7]	Description of HD in Tasmania indicating a common ancestor. No prevalence or incidence estimates.
Carter 1983[w8]	No estimates of incidence or prevalence of HD
Cchutani 1957[w9]	Description of cases of HD in the Punjab but no estimates of incidence or prevalence of HD.
Cendrowski 1964[w10]	No estimate of incidence or prevalence of HD
Critchley 1934[w11]	Only hospitalised patients committed under Mental Health Act. No population denominator
Cruz-Croke 1994[w12]	Appears to be a mixture of patients reported in the literature supplemented by own hospital data. We cant exclude the possibility of double counting.
Ekestern 2005[w13]	Austrian study of mortality rates of people with HD but no incidence or prevalence or data.
Gatto 2009[w14]	No estimate of incidence or prevalence of HD
Gatto 2010[w15]	No estimate of incidence or prevalence of HD.
Ginter 1999[w16]	No specific estimate of numbers of HD patients and no estimate of incidence or prevalence
Harper 1981[w17]	No original data on incidence or prevalence of HD.
Harvey 2003[w18]	HD patients confined to those aged <65 years with cognitive impairment. No population estimate.
Hecimovic 2002[w19]	Not a study of incidence
Hemminki 2006[w20]	Study of hospitalisation rates for HD but no population-based estimates of incidence or prevalence rates
Hendricks 2009[w21]	Study of probabilities of inheriting expanded CAG repeat but no estimates of incidence or prevalence rates.
Huifang 2010[w22]	Part review and part case-series. No prevalence data.

Husquinet 1985[w23]	Uses data from psychiatric registers and expresses prevalence in relation to live births.
Imaizumi 1993[w24]	Concerned solely with mortality rates based on death certification.
Korenyi 1973[w25]	No incidence or prevalence estimates.
Laccone 1999[w26]	Not an incidence or prevalence study
Lawal 2009[w27]	No data relating to incidence or prevalence of HD
Loy 2010[w28]	No original data; an estimate of the likely increase based on “population structure” since McCusker 2000.
Marx 1973[w29]	Study of genetic fitness. No estimate of prevalence or incidence
Mattsson 1985[w30]	No new data on incidence or prevalence from Mattsson 1974
Minski 1938[w31]	No estimate of incidence or prevalence of HD.
Morrison 2010[w32]	Provides a prevalence estimate for 2001 and full details were published in Morrison 2011 but no estimates of incidence
Paradisi 2008[w33]	No estimate of incidence or prevalence of HD
Pavoni 1990[w34]	Duplicate of incidence and prevalence (1971-1987) estimates by Govoni 1988
Petrin 1997[w35]	No specific estimates of the incidence and prevalence of HD.
Pramanik 2000[w36]	No estimate of prevalence or incidence of HD
Quarrell 2009[w37]	No original data on incidence or prevalence of HD
Raskin 2000[w38]	No incidence/prevalence estimates
Rubinsztein 1994[w39]	No original data on incidence or prevalence of HD
Saleem 2003[w40]	No original data on incidence or prevalence of HD
Scholefield 2007[w41]	No original data on incidence or prevalence of HD
Shaw 1982[w42]	No original data on incidence or prevalence of HD
Siesling 1997[w43]	No estimate of the prevalence/incidence of HD
Singer 1962[w44]	Not a population study. No estimate of incidence or prevalence of HD
Spillane 1937[w45]	No estimates of incidence or prevalence of HD
Squitieri 1994[w46]	No estimate of prevalence/incidence.
Whittier 1973[w47]	No estimate of prevalence but enumeration of the number of living patients in one institution (n=7) plus others (n=6) in the community.

Annex 5 (Web extra)
Further details of included studies

Study ID	Location	Incidence year(s)	Ages studied	Population size	Numbers of incident cases	Patient-years	Incidence per 1000,000 person-years (95%CIs)	Age of onset	Comments
Eastern Asia									
Chen 1968[6]	Guam	1960-1966	All ages	37,975	0	265,825	0.00 (0 to 13.9)	Not stated	
Chang 1994[26]	Hong Kong China	1984-1991	All ages	5,440,000	20	43,520,000	0.46 (0.28 to 0.71)*	37.6 (range 20 to 52)	
Chen 2010[9]	Taiwan	2000-2007	All ages	2001 = 22.4m<br>2002 = 22.5m<br>2003 = 22.6m<br>2004 = 22.7m<br>2005 = 22.8m<br>2006 = 22.9m<br>2007 = 23.0m	2001 = 11<br>2002 = 21<br>2003 = 27<br>2004 = 36<br>2005 = 31<br>2006 = 20<br>2007 = 19	2001 = 22.4m<br>2002 = 22.5m<br>2003 = 22.6m<br>2004 = 22.7m<br>2005 = 22.8m<br>2006 = 22.9m<br>2007 = 23.0m	2001 = 0.5 (0.2 to 0.9)*<br>2002 = 0.9 (0.5 to 0.9)*<br>2003 = 1.0 (0.7 to 2.0)*<br>2004 = 2.0 (1.0 to 2.0)*<br>2005 = 1.0 (0.9 to 2.0)*<br>2006 = 0.8 (0.5 to 1.0)*<br>2007 = 0.8 (0.4 to 1.0)*	Not stated	Also includes age specific rates
Australasia									
McCusker 2000[29]	NSW Australia	1991 & 1996	All ages	1991 = 5,732,031<br>1996 = 6,038,969	1991 = 26<br>1996 = 39	1991 = 5,732,031<br>1996 = 6,038,969	1991 = 4.5 (3.0 to 6.7)<br>1996 = 6.5 (4.6 to 8.8)	47.9 (SD 13.7)	
Europe									
Palo 1987[7]	Finland	1980s	All ages	4,900,000	2	4,900,000	0.2 to 0.4 (0.02 to 1.3)*	Not stated	Number of incident HD cases by back extrapolation = 1 or 2 depending on which incidence estimate used!
Govoni 1988[27]	Ferrara, Italy	1971-1987	All ages	377,006	1971-1975 = 3<br>1976-1980 = 1<br>1981-1987 = 3<br>1971-1987 = 7	6,032,096	1971-1975 = 1.5 (0.3 to 4.4)<br>1976-1980 = 0.5 (0.01 to 2.8)<br>1981-1987 = 1.1 (0.2 to 3.2)<br>1971-1987 = 1.1 (0.4 to 2.3)	Not stated	Patient years calculated by back extrapolation.
Ramos-Arroy 2005[28]	Navara & Basque, Spain	1994-2002	All ages	2,550,000	1994 to 2002 = 111	21,165,000	4.7 (4.5 to 6.3)*	43.7 (SD 15)	Population estimate by back extrapolation<br>My Cis (but need checking!)
Mercy 2008[8]	Cambridge UK	2000-2006	45 to 64 years	75,600	9	453,600	8.0 (2.0 to 23)	Not stated	Restricted age population

Sackley 2011[10]	United Kingdom	2004-2008	All ages	2004=2,840,202<br>2005=2,948,206<br>2006=2,977,044<br>2007=2,983,870<br>2008=2,964,386	2004 = 14<br>2005 = 23<br>2006 = 19<br>2007 = 15<br>2008 = 13	2004 = 2,840,202<br>2005 = 2,948, 206<br>2006 = 2,977,044<br>2007 = 2,983,870<br>2008 = 2,964,386	2004 = 7 (3 to 8)*<br>2005 = 7.8 (5.0 to 12)*<br>2006 = 6 (4 to 10)*<br>2007 = 5 (3 to 8)*<br>2008 = 4 (2 to 7)*	48.3 (SD14.)	Not sure whether the age at diagnosis relates to the incident cases only or includes prevalent ones too
Sveinsson 2012[11]	Iceland	1988-2007	All ages	2007=311,114	8	5,714,285	1.4 (0.6 to 2.8)*	51 (range 28-68)	Patient years calculated by back extrapolation
Douglas 2013[12]	United Kingdom	1990-2010	<21 years	1990 = 248,518<br>2010 = 1,167,683	12	17,142,857	0.70 (0.36 to 1.22)	Median = 15 (range 5 to 20)	
Current study	United KIngdom	1990-2010	>20 years	9,282,126	393	54,907,468	7.2 (6.5 to 7.9)	52 years (SD 16)	Also includes incidence estimates by year 1990-2010
North America									
Kokman 1994[30]	Minnesota USA	1950-1989	All ages	106,000	10	4,134,000	Definite = 3.0 (1.0 to 5.0)<br>Def + prob = 5.0 (3.0 to 9.0)	Not stated	Patient years calculated by back extrapolation.
Almqvist 2001[31]	British Columbia Canada	1996-1999	All ages	4,000,000	110	16,058,394	6.9 (5.7 to 8.3)*	46.9 (SD 13.7)	Author states population is “approx”. Patient years calculated by back extrapolation.

Annex 4 (As web extra)
References to excluded studies

w1. Adams P, Falek A. Arnold J. Huntington disease in Georgia: age at onset. American Journal of Human Genetics 1988; 43: 695-704.

w2. Aiyesimoju AB, Osuntokun BO, Badeemosi O, Adeuja AO. Hereditary neurodegenerative disorders in Nigerian Africans, Neurology 1984;34:361-362.

w3. Alonso M.E., Ochoa A., Boll M.-C., Sosa A.L., Yescas P., Lopez M., Macias R., Familiar I., Rasmussen A. Clinical and genetic characteristics of Mexican Huntington's disease patients. Movement Disorders 2009; 24: 2012-2015.

w4. Avila-Giron R. Medical and social aspects of Huntington's chorea in the state of Zulia. Advances in Neurology 1973; 1: 261-266

w5. Bayulkem F, Turek I. Huntington's chorea in Turkey. Psychiatry Quarterly 1961; 35: 358-360.

w6. Bell J. Nervous diseases and muscular dystrophies. Part 1: Huntington's Chorea, In; Treasury of Human Inheritance. London: Cambridge University press,1934;4:1-29.

w7. Brothers CRD. The history and incidence of Huntington's disease in Tasmania. Proc Roy Soc Aust Coll Phys 1949; 4: 48-50.

w8. Carter C, Evans KA, Baraitser M. Effect of genetic counselling on the prevalence of Huntington's chorea. BMJ 1983; 286: 281-283.

w9. Cchutani PN. Huntington’s chorea in India. Journal of the Indian Medical Association 1957; 29: 156-157.

w10. Cendrowski W. Niektore dane o geografii lasawicy dziedzicznej. Neur. Neurochir. Psychiat. Pol. 1964;14:63-66.

w11. Critchley M. Huntington’s chorea and East Anglia. Journal of State Medicine 1934; 42: 575-587.

w12. Cruz-Coke R, Moreno RS. Genetic epidemiology of single gene defects in Chile. Journal of Medical Genetics 1994; 31: 702-706.

w13. Ekestern E, Lebhart G. Long-term monitoring of the mortality trend of Huntington's disease in Austria. European Journal of Epidemiology 2005; 20: 169-172.

w14. Gatto E.M., Parisi V.L., Persi G.G., Etcheverry J.L., Leiguarda F., Varela V. Huntington's disease in a cohort from Argentina. Journal of Neurology. Conference: 19th Meeting of the European Neurological Society Milan Italy. Conference Start: 20090620 Conference End: 20090624. Conference Publication: (var.pagings). 256 (pp S222), 2009.

w15. Gatto E.M., Parisi V., Persi G.G., Etcheverry J.L., Leiguarda F., Lpez A.P., Varela V. Estimation of prevalence and molecular characteristics among Huntington's disease patients of Argentina. Movement Disorders. Conference: 14th International Congress of Parkinson's Disease and Movement Disorders Buenos Aires Argentina. Conference Start: 20100613 Conference End: 20100617.

w16. Ginter EK. Epidemiology of hereditary disorders in Russia’s populations. 1999

w17. Harper PS. Tyler A. Smith S. Jones P. Newcombe RG. McBroom V. Decline in the predicted incidence of Huntington's chorea associated with systematic genetic counselling and family support. Lancet. 1981; 2: 411-3.

w18. Harvey RJ, Skelton-Robinson M, Rossor MN. The prevalence and causes of dementia in people under the age of 65 years. Journal of Neurology Neurosurgery and Psychiatry 2003; 74: 1206-1209.

w19. Hecimovic S., Klepac N., Vlasic J., Vojta A., Janko D., Skarpa-Prpic I., Canki-Klain N., Markovic D., Bozikov J., Relja M., Pavelic K. Genetic background of Huntington disease in Croatia: Molecular analysis of CAG, CCG, and Delta2642 (E2642del) polymorphisms. Human Mutation 2002; 20: 233.

w20. Hemminki K, Sundqvist K, Li X. Famial risks for main neurological diseases in siblings based on hospitalizations in Sweden. Twin Research & Human Genetics 2006; 9: 580-586.

w21. Hendricks AE. Latourelle JC. Lunetta KL. Cupples LA. Wheeler V. MacDonald ME. Gusella JF. Myers RH. Estimating the probability of de novo HD cases from transmissions of expanded penetrant CAG alleles in the Huntington disease gene from male carriers of high normal alleles (27-35 CAG). American Journal of Medical Genetics. Part A. 2009; 149A:1375-1381.

w22. Huifang S., Pei Z., Burgunder J.-M. Huntington's disease in China. Journal of Neurology, Neurosurgery and Psychiatry. Conference: European Huntington's Disease Network, EHDN Annual Meeting 2010 Prague Czech Republic. Conference Start: 20100903 Conference End: 20100905. Conference Publication: (var.pagings). 81 (pp A21), 2010.

w23. Husquinet H. Huntington's chorea, the Liege experience. J Genet Hum 1985; 33: 10-18.

w24. Imaizumi Y. Mortality rates for Huntington's disease in Japan, 1969-1972: geographical variations. In Intractable Neurological Disorders, Human Genome Research and Society. Proceedings of the Third International Bioethics Seminar in Fukui, 19-21 November 1993, pp 77-80.

w25. Korenyi CK, Whittier JR. The juvenile for of Huntington's chorea: its prevalence and other observations. Advances in Neurology 1973; ! 237-243.

w26. Laccone F., Engel U., Holinski-Feder E., Weigell-Weber M., Marczinek K., Nolte D., Morris-Rosendahl D.J., Zuhlke C., Fuchs K., Weirich-Schwaiger H., Schluter G., Von Beust G., Vieira-Saecker A.M.M., Weber B.H.F., Riess O. DNA analysis of Huntington's disease: Five years of experience in Germany, Austria, and Switzerland. Neurology 1999; 53: 801-806.

w27. Lawal O., Gbadebo A.E. Inadequate knowledge and awareness on Huntington's disease: The risk factors for increased Huntington's disease incidence in Nigeria. Clinical Genetics. Conference: 2009 World Congress on Huntington's Disease Vancouver, BC Canada. Conference Start: 20090912 Conference End: 20090915.

w28. Loy C.T., Lownie A., McCusker E. Huntington's disease. The Lancet. 376 (9751) (pp 1463), 2010.

w29. Marx RN. Huntington's chorea in Minnesota. Advances in Neurology 1973; ! 237-243.

w30. Mattsson B, Ottosson J-O. Huntington's sjukdom I uppdaterad register. Lakartidnungen 1985; 82: 1177.

w31. Minski L, Guttman E. Huntington's Chorea: a study of 34 families. Journal of Mental Science 1938; 84: 21-96.

w32. Morrison P.J. Accurate prevalence and uptake of testing for Huntington's disease. The Lancet Neurology. 9 (12) (pp 1147), 2010.

w33. Paradisi I, HernandezA, Arias S. Huntington disease mutation in Venezuela:age of onset, haplotype analyses, and geographic aggregation. Journal of Human Genetics 2008; 53: 127-135.

w34. Pavoni M., Granieri E., Govoni V., Pavoni V., Del Senno L., Mapelli G. Epidemiologic approach to Huntington's disease in Northern Italy (Ferrara area). Neuroepidemiology 1990; 9:306-314.

w35. Petrin AN, Perepelov AV, Nurbaev SD, Balanovskaya EV, Sitnikov VF, Inzemtseva VS, Rudenskaya GE. Hereditary nervous system diseases in Mordovia. Genetika 1997; 33: 836-843.

w36. Pramanik S., Basu P., Gangopadhaya P.K., Sinha K.K., Jha D.K., Sinha S., Das S.K., Maity B.K., Mukherjee S.C., Roychoudhuri S., Majumder P.P., Bhattacharyya N.P. Analysis of CAG and CCG repeats in Huntingtin gene among HD patients and normal populations of India. European Journal of Human Genetics 2000; 8: 678-682.

w37. Quarrell O. Living with juvenile HD. Clinical Genetics. Conference: 2009 World Congress on Huntington's Disease Vancouver, BC Canada. Conference Start: 20090912 Conference End: 20090915. Conference Publication: (var.pagings). 76 (pp 3), 2009.

w38. Raskin S., Allan N., Teive H.A., Cardoso F., Haddad M.S., Levi G., Boy R., Lerena Junior J., Sotomaior V.S., Janzen-Duck M., Jardim L.B., Fellander F.R., Andrade L.A. Huntington disease: DNA analysis in Brazilian population. Arquivos de neuro-psiquiatria. 2000; 58: 977-985.

w39. Rubinsztein DC. Amos W. Leggo J. Goodburn S. Ramesar RS. Old J. Bontrop R. McMahon R. Barton DE. Ferguson-Smith MA. Mutational bias provides a model for the evolution of Huntington's disease and predicts a general increase in disease prevalence. Nature Genetics 1994: 7: 525-30,

w40. Saleem Q., Roy S., Murgood U., Saxena R., Verma I.C., Anand A., Muthane U., Jain S., Brahmachari S.K. Molecular analysis of Huntington's disease and linked polymorphisms in the Indian population. Acta Neurologica Scandinavica 2003; 108: 281-286.

w41. Scholefield J, Greenberg J.A common SNP haplotype provides molecular proof of a founder effect of Huntington disease linking two South African populations. European Journal of Human Genetics (2007) 15, 590 – 595

w42. Shaw M., Caro A. The mutation rate to Huntington's chorea. Journal of Medical Genetics. 1982; 19: 161-167), 1982.

w43. Siesling S., Vegter-Van Der Vlis M., Roos R.A.C. Juvenile Huntington disease in the Netherlands. Pediatric Neurology 1997; 17: 37-43.

w44. Singer K. Huntington’s disease in the Chinese. BMJ 19632;ii:1311-1312

w45. Spillane J, Phillips R. Huntington’s chorea in South Wales. Quarterly Journal of Medicine 1937; 24: 403-423

w46. Squitieri F., Andrew S.E., Goldberg Y.P., Kremer B., Spence N., Zeisler J., Nichol K., Theilmann J., Greenberg J., Goto J., Kanazawa I., Vesa J., Peltonen L., Almqvist E., Anvret M., Telenius H., Lin B., Napolitano G., Morgan K., Hayden M.R. DNA

haplotype analysis of Huntington disease reveals clues to the origins and mechanisms of CAG expansion and reasons for geographic variations of prevalence. Human Molecular Genetics 1994; 3: 2103-2114.

w47. Whittier J.R., Korenyi C., Sutanto D. Prevalence of Huntington chorea. JAMA 1973; 226: 1465-1466.
